# Expression Profiles and Functional Characterization of Chemosensory Protein 15 (HhalCSP15) in the Brown Marmorated Stink Bug *Halyomorpha halys*

**DOI:** 10.3389/fphys.2021.721247

**Published:** 2021-09-06

**Authors:** Zehua Wang, Fan Yang, Ang Sun, Shuang Shan, Yongjun Zhang, Shanning Wang

**Affiliations:** ^1^Beijing Key Laboratory of Environment Friendly Management on Fruit Diseases and Pests in North China, Institute of Plant and Environment Protection, Beijing Academy of Agriculture and Forestry Sciences, Beijing, China; ^2^State Key Laboratory for Biology of Plant Diseases and Insect Pests, Institute of Plant Protection, Chinese Academy of Agricultural Sciences, Beijing, China

**Keywords:** *Halyomorpha halys*, chemosensory protein, expression profile, fluorescence binding assay, molecular docking, electroantennography

## Abstract

Chemosensory proteins (CSPs) have been identified in the sensory tissues of various insect species and are believed to be involved in chemical communication in insects. However, the physiological roles of CSPs in *Halyomorpha halys*, a highly invasive insect species, are rarely reported. Here, we focused on one of the antennal CSPs (*HhalCSP15*) and determined whether it was involved in olfactory perception. Reverse transcription PCR (RT-PCR) and quantitative real-time PCR (qRT-PCR) analysis showed that *HhalCSP15* was enriched in nymph and male and female adult antennae, indicating its possible involvement in the chemosensory process. Fluorescence competitive binding assays revealed that three of 43 natural compounds showed binding abilities with HhalCSP15, including β-ionone (*K_i_*=11.9±0.6μM), *cis*-3-hexen-1-yl benzoate (*K_i_*=10.5±0.4μM), and methyl (2*E*,4*E*,6*Z*)-decatrienoate (*EEZ*-MDT; *K_i_*=9.6±0.8μM). Docking analysis supported the experimental affinity for the three ligands. Additionally, the electrophysiological activities of the three ligands were further confirmed using electroantennography (EAG). *EEZ*-MDT is particularly interesting, as it serves as a kairomone when *H. halys* forages for host plants. We therefore conclude that HhalCSP15 might be involved in the detection of host-related volatiles. Our data provide a basis for further investigation of the physiological roles of CSPs in *H. halys*, and extend the olfactory function of CSPs in stink bugs.

## Introduction

Many insects rely on their sense of smell to locate food sources, search for mating partners, select oviposition sites, and avoid predators (Metcalf and Kogan, 1987; Takken, 1991; Bruce et al., 2005; Ebrahim et al., 2015). The detection of olfactory signals in insects is performed by olfactory receptor neurons located in olfactory sensilla, which are present on the antennae and other head appendices. Olfactory sensilla are perforated by numerous pores, forming a hollow structure filled with aqueous lymph that harbors the dendritic branches of olfactory receptor neurons and contains abundant small soluble binding proteins (Maida et al., 1993; Steinbrecht, 1997). In the initial stage of olfactory reception, odorants enter the olfactory sensillum cavity through pore canals, and are transported by soluble binding proteins to the olfactory receptor on dendrite membranes (Vogt and Riddiford, 1981; Kaissling, 2009; Leal, 2013).

Odorant binding proteins (OBPs) and chemosensory proteins (CSPs) are the two main types of soluble binding proteins. A large body of evidence from different approaches has extensively documented that OBPs bind to pheromones and odorants, with different degrees of affinity and selectivity for different OBPs (Pelosi et al., 2014; Zhang et al., 2017; Huang et al., 2018; Wang et al., 2020; Rihani et al., 2021). In several instances, it was demonstrated that OBPs are involved not just in detecting olfactory stimuli, but also in modulating stimulus sensitivity in the olfactory system (Xu et al., 2005; Larter et al., 2016; Gonzalez et al., 2020). CSPs may perform functions similar to OBPs in the olfactory system.

Insect CSPs are known as olfactory segment D (OS-D) or A-10 before being named as CSPs because of their high expression in chemosensory organs (McKenna et al., 1994; Pikielny et al., 1994; Angeli et al., 1999). CSPs are smaller than OBPs (100–120 residues) and bear no sequence similarity to OBPs. They present a motif of four conserved cysteines linked by disulfide bridges (Angeli et al., 1999). The three-dimensional (3D) structure of CSP protein is composed of six α-helices that define a hydrophobic cavity (Lartigue et al., 2002). As an olfactory protein, CSPs have been studied in Lepidoptera, Hemiptera, Hymenoptera, and Coleoptera (Gu et al., 2012; Sun et al., 2014; Peng et al., 2017; Fu et al., 2020). CSPs in the alfalfa plant bug *Adelphocoris lineolatus* have been implicated in mediating host recognition (Gu et al., 2012; Sun et al., 2015). However, numerous studies have shown that the expression of CSPs is not restricted to the antennae, and many CSP genes are expressed in other parts of the insect body, with functions different from olfaction. In non-olfactory tissues, they are believed to be involved in development, pheromone delivery, nutrient absorption, insecticide resistance, vision, and immune response (Nomura et al., 1992; Forêt et al., 2007; Maleszka et al., 2007; Bos et al., 2010; Xuan et al., 2015; Pelosi et al., 2017).

The brown marmorated stink bug *Halyomorpha halys* (Stål; Hemiptera: Pentatomidae), which is native to Asia, is an invasive pest that in the last few decades has rapidly spread globally, including in the United States, Canada, and Europe (Hoebeke and Carter, 2003; Wermelinger et al., 2008; Leskey and Nielsen, 2017). In its native and introduced range, *H. halys* feeds on more than 100 crops, and it has become a destructive pest of many crops in the world (Lee et al., 2013; Haye et al., 2015; Kriticos et al., 2017; Leskey and Nielsen, 2017). Being an important invasive pest worldwide, there are many studies examining the chemical ecology of *H. halys* (Khrimian et al., 2014; Harris et al., 2015; Leskey et al., 2015; Weber et al., 2017). In *H. halys*, antennal transcriptomic approaches have already led to the identification of 17 CSP genes (Sun et al., 2020), which now await functional characterization.

In this study, to examine the potential role of one antennal CSP (*HhalCSP15*) in olfaction perception, extensive expression profiling of *HhalCSP15* transcripts was performed using semi-quantitative reverse transcription PCR (RT-PCR) and quantitative real-time PCR (qRT-PCR) methods among different tissues in nymph and male and female adult stages. We further expressed HhalCSP15 *in vitro* and determined its binding affinities for 43 volatiles in fluorescence binding assays. Further, homology modeling and molecular docking were applied for predicting the key amino acids of HhalCSP15 that bind candidate ligands. In addition, electrophysiological activities of HhalCSP15 ligands were confirmed using electroantennography (EAG) recordings.

## Materials and Methods

### Insect Culture, Tissue Collection, Total RNA Isolation, and cDNA Synthesis

Overwintering *H. halys* adults were collected from Beijing Xishan National Forest Park, Beijing, China. The laboratory colony was established in plastic containers (20cm×13cm×8cm), which were maintained at 25±1°C, 60±10% relative humidity, and a 16L:8D photoperiod. The adults and nymphs were reared on green beans. Different tissues from third instar nymphs (antennae, mouthparts, heads, thoraxes, abdomens, and legs) and 1- to 3-day-old female and male adults (antennae, mouthparts, heads, thoraxes, abdomens, legs, and wings) were collected. All tissues were immediately frozen in liquid nitrogen and stored at −80°C until RNA isolation.

Total RNA was extracted from different tissues using TRIzol reagent (Invitrogen, Carlsbad, CA, United States) following the manufacturer’s protocol. The integrity and quantity of RNA samples were checked using 1.2% agarose gel electrophoresis and a NanoPhotometer N60 (Implen, München, Germany), respectively. cDNA from different tissues was synthesized from 2μg of RNA using the Fast Quant RT kit with gDNase (Tiangen, Beijing, China) for gene cloning and tissue expression pattern analyses.

### Verification of the *HhalCSP15* Sequence by Cloning and Sequencing

Gene-specific primers were designed to clone the open reading frame (ORF) of *HhalCSP15*. PCR was performed using one unit of KOD DNA polymerase (Taihe, Beijing, China) and 200ng cDNA under the following conditions: denaturation at 94°C for 2min, followed by 30cycles of denaturation at 94°C for 20s, annealing at 55°C for 30s, and extension at 68°C for 1min. The final extension step was at 68°C for 5min. The PCR products were cloned into a pCloneEZ-Blunt vector (Taihe, Beijing, China), and cloned products were sequenced using the M13 primer.

### Reverse Transcription PCR

The expression of *HhalCSP15* in different tissues of nymphs and male and female adults was analyzed by RT-PCR using Taq DNA polymerase (Biomed, Beijing, China). Each PCR volume (25μl) contained 200ng of cDNA from different tissues and was used as a template. The following cycling conditions were applied: 94°C for 4min, and for the subsequent 30cycles: 94°C for 30s, 55°C for 30s, and 72°C for 45s. The final extension step was at 72°C for 5min. The elongation factor 1-α (EF-1α, XM_014414739.2) was employed to assess the cDNA integrity for all samples. The amplification products were checked on 1.2% agarose gels. For each gene, one amplification product was sequenced to confirm its identity. The gene-specific primers were designed using Primer 3^1^ and are listed in Supplementary Table S1.

### Quantitative Real-Time PCR

The relative transcript abundance of *HhalCSP15* in the antennae, mouthparts, and legs of nymphs and male and female adults was determined by qRT-PCR. qRT-PCR was conducted using an ABI Prism 7500 System (Applied Biosystems, Carlsbad, CA, United States) and SYBR Green SuperReal PreMix Plus (TianGen, Beijing, China). Each qRT-PCR reaction was conducted in a 20μl reaction mixture containing 10μl of 2× SuperReal PreMix Plus, 1μl (200ng) of sample cDNA, 0.4μl of 50× ROX Reference Dye, and 6.1μl of sterilized ultrapure water. Each qRT-PCR experiment was performed using three biological replicates, and each biological replicate was assessed three times. The ubiquitin conjugation factor E4 A (Ubiquitin, XM_014429239.2) and EF-1α were used as endogenous controls to normalize the target gene expression and correct for any sample-to-sample variation.

The comparative 2^−∆∆CT^ method (Livak and Schmittgen, 2001) was used to calculate the relative transcript levels in each tissue. The primers of the target and reference genes are listed in Supplementary Table S1. The specificity of each primer set was validated by melting curve analysis, and the efficiency was calculated by analyzing the standard curves with a 5-fold cDNA dilution series. The comparative analyses of *HhalCSP15* expression among different tissues and developmental stages were conducted with one-way ANOVA, followed by Tukey’s honestly significant difference (HSD) test using SPSS Statistics 18.0 (SPSS Inc., Chicago, IL, United States).

### Expression and Purification of Recombinant HhalCSP15

*HhalCSP15* was PCR-amplified using gene-specific primers (Supplementary Table S1). The PCR products were first subcloned into a T vector (Taihe, Beijing, China) and then into the bacterial expression vector pET30a (+; Novagen, Madison, WI, United States) between the NdeI and XhoI sites, and were verified by sequencing. The plasmids containing the correct insert were transformed into BL21 (DE3) competent cells. The protein was expressed in LB at 18°C for 16h through induction with 1mM of isopropyl-β-D-thiogalactopyranoside (IPTG). The cultures were harvested by centrifugation and resuspended in a 50mM Tris buffer (pH 7.4). After sonication and centrifugation, the recombinant proteins, which were mainly present in the supernatant, were purified by a standard Ni column (GE Healthcare, Waukesha, WI, United States). The His-tag was removed using a recombinant enterokinase (Novagen) following the manufacturer’s protocol. Purified HhalCSP15 was dialyzed in the Tris buffer, and its concentration was determined by the Bradford method (Bradford, 1976).

### Fluorescence Competitive Binding Assays

The binding abilities of HhalCSP15 to 43 volatiles were measured on an F-380 fluorescence spectrophotometer (Tianjin, China) using 10-nm slits and a 1-cm light path. As the fluorescent probe, N-phenyl-1-naphthylamine (1-NPN) was excited at the wavelength of 337nm, and emission spectra were recorded between 390 and 530nm. To measure the affinity of 1-NPN to the HhalCSP15 protein, a 2-μM solution of purified protein in 50-mM Tris-HCl at pH 7.4 was titrated with aliquots of 1-mM 1-NPN dissolved in methanol to final concentrations ranging from 2 to 16μM.

Competitive binding was measured by titration of the solution of both HhalCSP15 protein and 1-NPN at a concentration of 2μM by adding aliquots of 1-mM methanol solution of ligand to final concentrations of 2–20μM. Dissociation constants of the competitors were calculated by the equation *K_i_*=IC_50_/(1+[1-NPN]/K_1-NPN_), where IC_50_ is the concentration of ligands halving the initial fluorescence value of 1-NPN, [1-NPN] is the free concentration of 1-NPN, and K_1-NPN_ is the dissociation constant of the HhalCSP15/1-NPN complex. The experiments were performed in triplicate, excepting the ligands that did not show significant binding, which were analyzed in single experiments.

### 3D Structure Modeling and Molecular Docking

A suitable template for 3D modeling was identified using a sequence of similar searches at PSI-BLAST against sequences from the Protein Data Bank.^2^ Because of high sequence similarity with the HhalCSP15 protein, the high-resolution structure of a CSP from *Mamestra brassicae* (PDB: 1N8V) was selected for homology modeling using MODELLER 9.25.^3^ The structure refinement of the protein model was achieved by energy minimization *via* molecular dynamic simulation (MD) using GROMACSv5.0.7 with AMBER99SB force field (Abraham et al., 2015). A Ramachandran plot was performed to evaluate the rationality of the established 3D model using the online tool PROCHECK.^4^ The ligands were 3D optimized in ChemDraw 3D (PerkinElmer, Waltham, MA, United States) and refined with energy minimization. Ligands were docked to the model of HhalCSP15 using Autodock Vina.^5^ The best binding modes were selected according to the lowest free binding energy (kcalmol^−1^). The docked models of proteins interacting with ligands were displayed using PYMOL.^6^

### Electrophysiological Recordings

The antennal responses of *H. halys* to the three ligands of HhalCSP15 were evaluated using conventional EAG methods as described in previous study (Wang et al., 2020). Briefly, antennae of the third instar nymphs and 1- to 3-day old male and female adults were dissected, and a few terminal segments at the distal end were excised. The treated antennae were attached to electrode holders with electrode gel. Ten microliters of tested chemicals (100μg/μl, diluted in paraffin oil) were applied to filter paper strips (1.0cm×2.0cm) and inserted into a glass Pasteur pipette as a cartridge. The test cartridge was connected to a stimulus controller (CS-55; Syntech, Kirchzarten, Germany) that generated a 0.5s stimulus at 30s intervals, with a constant flow of 10ml/s. The signals generated by the antennae were recorded using EAG Pro (Syntech). A blank stimulus (solvent control) was presented before testing the compound. For each compound, EAG responses were recorded from eight antennae of different insects. The EAG responses elicited by the test odor stimuli were corrected by subtracting EAG response from the solvent control. The corrected EAG data were statistically analyzed using ANOVA followed by Tukey’s HSD test.

## Results

### Sequence Analysis of *HhalCSP15*

The nucleotide sequence of *HhalCSP15* was verified by molecular cloning and sequencing. Analysis of the *HhalCSP15* sequence revealed full-length ORFs consisting of 366 nucleotides that encode 121 amino acid residues. At its *N*-terminus, HhalCSP15 is predicted to contain a signal peptide consisting of 19 amino acid residues (Supplementary Figure S1). The predicted molecular weight of HhalCSP15 protein was 11.91kDa, and the isoelectric point was 7.76. HhalCSP15 had the typical four-cysteine signature and fit the motif pattern of C1-X6-8-C2-X16-21-C3-X2-C4 of insect CSPs (Zhou et al., 2006).

### Expression Profiles of *HhalCSP15*

**Figure 1:**
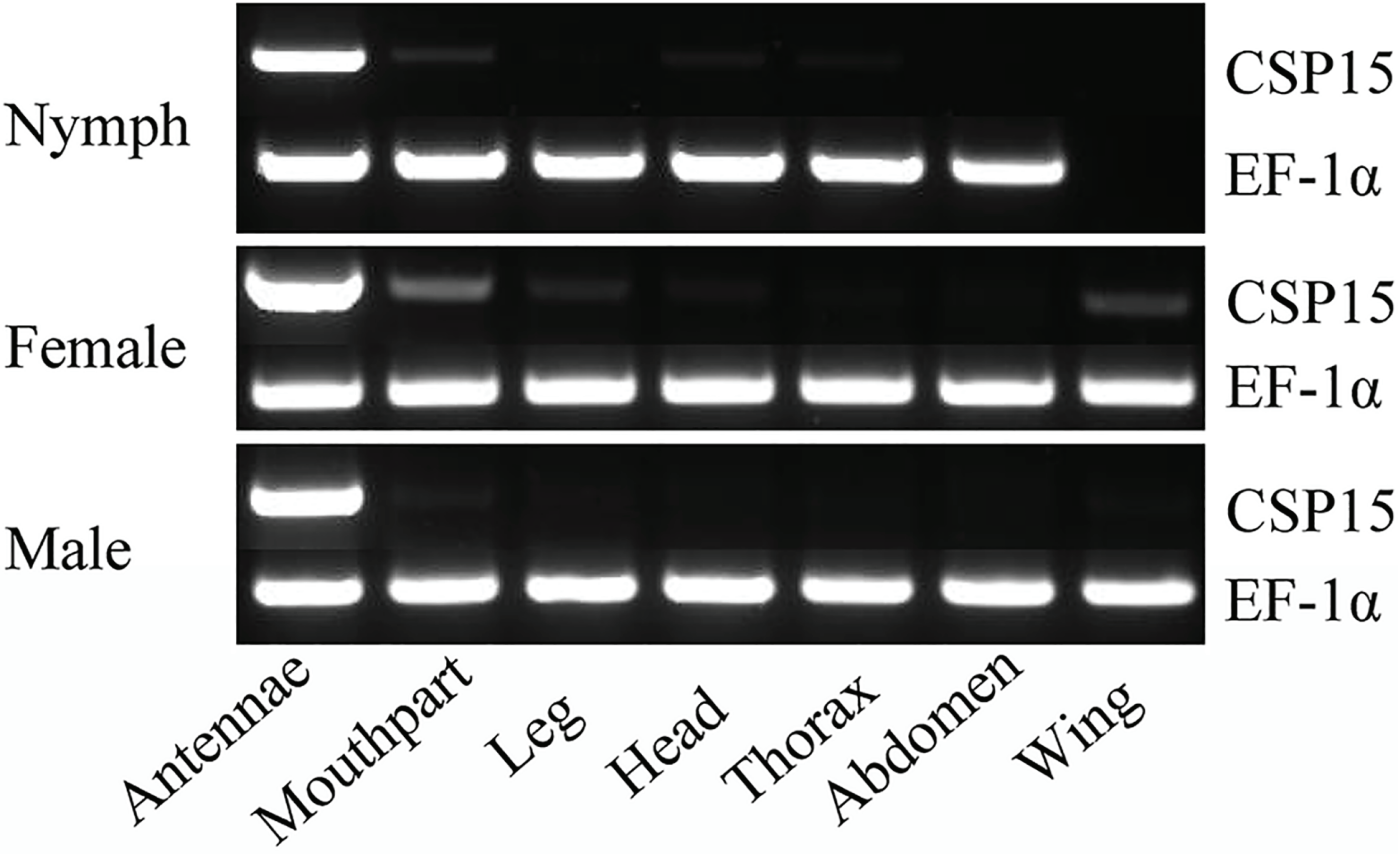
Tissue-specific expression of *HhalCSP15* in different stages (nymph and male and female adult) measured by reverse transcription PCR (RT-PCR). Elongation factor 1-α (EF-1α) was used as a control gene.

We used RT-PCR to analyze the tissue-specific expression of the *HhalCSP15* transcript in different nymph and adult tissues. The EF-1α gene was constitutively expressed in all tissues, thereby providing a stable control for the integrity of the cDNA templates (Figure 1). *HhalCSP15* was detected specifically in the antennae of nymph and male and female adult, although minor bands were detected in other tissues, such as mouthpart and leg (Figure 1).

**Figure 2:**
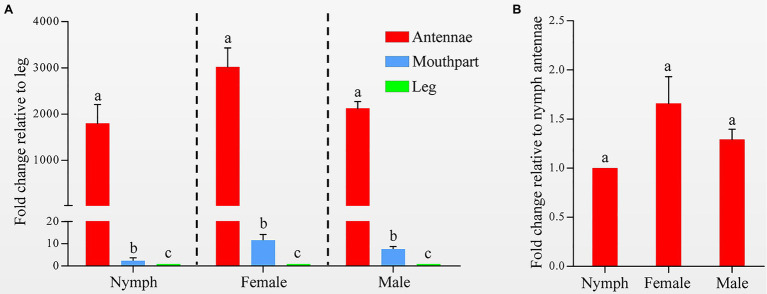
**(A)** Expression profiles of the *HhalCSP15* gene in different tissues (antennae, mouthpart, and leg) measured by quantitative real-time PCR (qRT-PCR). The fold changes are relative to the transcript levels in the leg. **(B)** Relative transcript levels of the *HhalCSP15* gene in the antennae at different stages. The fold changes are relative to the transcript levels in the antennae of nymph. Reference genes: EF-1α and Ubiquitin. The error bar and different letters represent the SE and significant differences, respectively (*p*<0.05).

Quantitative real-time PCR was used to measure the *HhalCSP15* transcript levels in different tissues. The *HhalCSP15* transcript was expressed significantly higher in the antennae than in other tissues; it was approximately 1,803, 3,023, and 2,130 times higher in the antennae than in the leg of nymphs and female and male adults, respectively. The expression of the *HhalCSP15* gene was also detected in the mouthpart at 11.6-, 7.6-, and 2.4-fold higher than the leg of nymphs and female and male adults, respectively (Figure 2A). Furthermore, the expression levels were approximately 1.7- and 1.3-fold higher in the female and male adult antennae than in the nymph antennae (Figure 2B).

### Binding Characteristic of Recombinant HhalCSP15

**Figure 3:**
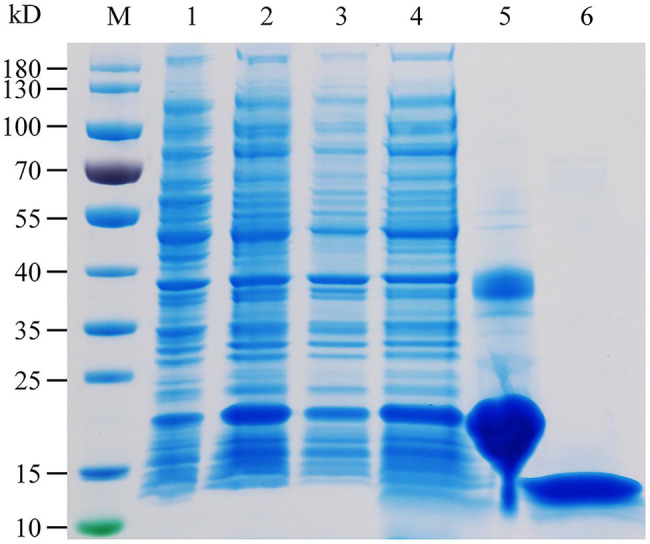
SDS-PAGE analysis of the recombinant HhalCSP15. M: molecular weight markers, 1: cell pellet before induction with IPTG, 2: cell pellet after induction, 3: pellet after sonication, 4: supernatant after sonication, 5: protein purified by affinity chromatography, and 6: purified protein after digestion with enterokinase.

The specific expression of HhalCSP15 in the antennae of nymphs and adults suggests that HhalCSP15 is potentially involved in peripheral olfactory reception for *H. halys*. To screen the putative ligands for HhalCSP15, we first expressed HhalCSP15 in a bacterial system. The protein was purified by affinity chromatography on Ni columns and was then used for ligand-binding experiments. The size and purity of the recombinant protein were examined by SDS–PAGE (Figure 3).

**Figure 4:**
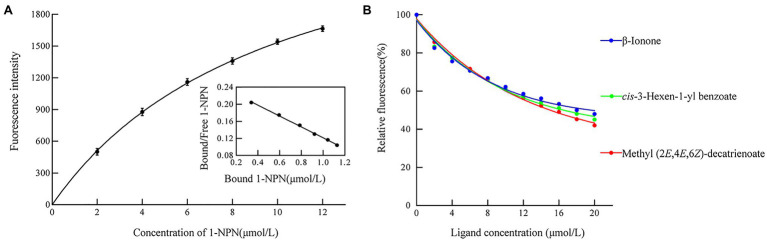
Binding properties of recombinant HhalCSP15. **(A)** Binding curve and Scatchard plot of N-phenyl-1-naphthylamine (1-NPN) to HhalCSP15. **(B)** Binding curves of HhalCSP15 to candidate ligands.

We measured the protein affinity to 43 volatile compounds in competitive binding experiments using 1-NPN as a fluorescent probe. First, the affinity constant was measured for HhalCSP15 to 1-NPN (Figure 4A). HhalCSP15 binds reversibly to 1-NPN with a dissociation constant of 9.36μM, which indicates that 1-NPN is a suitable fluorescent reporter. HhalCSP15 displayed a relatively specific binding spectrum; of the 43 tested odorants, only three compounds showed binding affinities for HhalCSP15: β-ionone, *cis*-3-hexen-1-yl benzoate, and methyl (2*E*,4*E*,6*Z*)-decatrienoate (*EEZ*-MDT), which had binding affinities of 11.9±0.6, 10.5±0.4, and 9.6±0.8μM, respectively (Figure 4B; Table 1).

**Table 1:** Binding affinities of all tested ligands to HhalCSP15.

Ligands	Source	CAS number	Purity (%)	IC_50_ (μM)	*K_i_* (μM)
1-Hexanol	TCI	111–27-3	>98.0	75.8	-
*cis*-3-Hexen-1-ol	TCI	928–96-1	>97.0	82.4	-
1-Octen-3-ol	TCI	3,391–86-4	>98.0	78.2	-
1-Octanol	TCI	111–87-5	>99.0	72.0	-
Hexanal	TCI	66–25-1	>98.0	78.9	-
Nonanal	TCI	124–19-6	>95.0	81.1	-
n-Octanal	TCI	124–13-0	>98.0	82.2	-
Decanal	TCI	112–31-2	>97.0	61.0	-
*trans*-2-Hexenal	TCI	6,728–26-3	>97.0	71.8	-
*trans*-2-Heptenal	TCI	18,829–55-5	>95.0	70.5	-
*trans*-2-Decenal	TCI	3,913–81-3	>93.0	48.5	-
*trans*-2-Octenal	TCI	2,548–87-0	>96.0	45.9	-
Benzaldehyde	TCI	100–52-7	>98.0	67.0	-
Octane	TCI	111–65-9	>97.0	79.2	-
Decane	TCI	124–18-5	>99.0	71.7	-
Undecane	TCI	1,120–21-4	>99.0	69.0	-
Dodecane	TCI	112–40-3	>99.0	88.4	-
Tridecane	TCI	629–50-5	>99.0	86.4	-
Methyl salicylate	TCI	119–36–8	>99.0	84.6	-
*cis*-3-Hexen-1-yl benzoate	TCI	25,152–85-6	>99.0	16.8	10.5±0.8
Methyl benzoate	TCI	93–58-3	>99.0	68.6	-
Isobornyl Acetate	TCI	125–12-2	>99.0	81.3	-
Hexyl butyrate	TCI	2,639–63-6	>98.0	61.9	-
*trans*-2-Hexenyl acetate	TCI	2,497–18-9	>97.0	75.2	-
Hexyl acetate	TCI	142–92-7	>99.0	83.9	-
*cis*-3-Hexenyl acetate	TCI	3,681–71-8	>97.0	77.2	-
*cis*-3-Hexenyl Isovalerate	TCI	35,154–45-1	>98.0	67.1	-
Methyl (2*E*,4*E*,6*Z*)-decatrienoate	Codow	51,544–64-0	>95%	15.3	9.6±0.4
2-Hexanone	TCI	591–78-6	>98.0	85.1	-
4'-Ethylacetophenone	TCI	937–30-4	>97.0	71.0	-
β-Ionone	TCI	14,901–07-6	>95.0	19.0	11.9±0.6
(−)-β-Pinene	TCI	18,172–67-3	≥94.0	70.7	-
Myrcene	Macklin	123–35-3	≥90.0	78.5	-
(+)-Limonene	TCI	5,989–27-5	>95.0	62.8	-
Nerolidol	TCI	7,212–44-4	>97.0	105.0	-
Ocimene	Sigma	13,877–91-3	≥90.0	69.0	-
β-Caryophyllene	TCI	87–44-5	>90.0	141.4	-
Linalool	TCI	78–70-6	>96.0	79.1	-
1,8-Cineole	TCI	470–82-6	>99.0	90.1	-
Citral	TCI	5,392–40-5	>96.0	66.4	-
(−)-Citronellal	TCI	5,949–05-3	>96.0	84.3	-
Eugenol	TCI	97–53-0	>99.0	111.7	-
Phenylacetonitrile	TCI	140–29-4	>98.0	78.4	-

IC_50_, concentration of ligand halving the initial fluorescence intensity; K_i_, dissociation constant; We consider the HhalCSP15 proteins had no binding with the tested ligands if the IC_50_ values>20μM and K_i_ values were not to be calculated and are represented as “-.”

### Protein Structure Prediction and Molecular Docking

To support the results of our ligand binding assay and provide insight into the mechanism of HhalCSP15 interaction with ligands, molecular docking of the three ligands with HhalCSP15 was performed. The best model for HhalCSP15 (Supplementary Figure S2) was obtained using the crystal structure of the CSP from *M. brassicae* (PDB: 1N8V, 42% identity) as a template. The protein model was subjected to a 50ns MD simulation to energy minimize and stabilize the protein. The structural stability of the protein was measured by evaluating root mean square deviation (RMSD) and root mean square fluctuation (RMSF; Supplementary Figure S3). A Ramachandran plot was employed to estimate the rationality of the predicted protein structure. It revealed that 93.5% of the residues were in the most favored allowed region, 6.5% of the residues were in the additional allowed region, and none were in the disallowed region (Supplementary Figure S4), suggesting that the predicted model of HhalCSP15 is reasonable and reliable.

**Figure 5:**
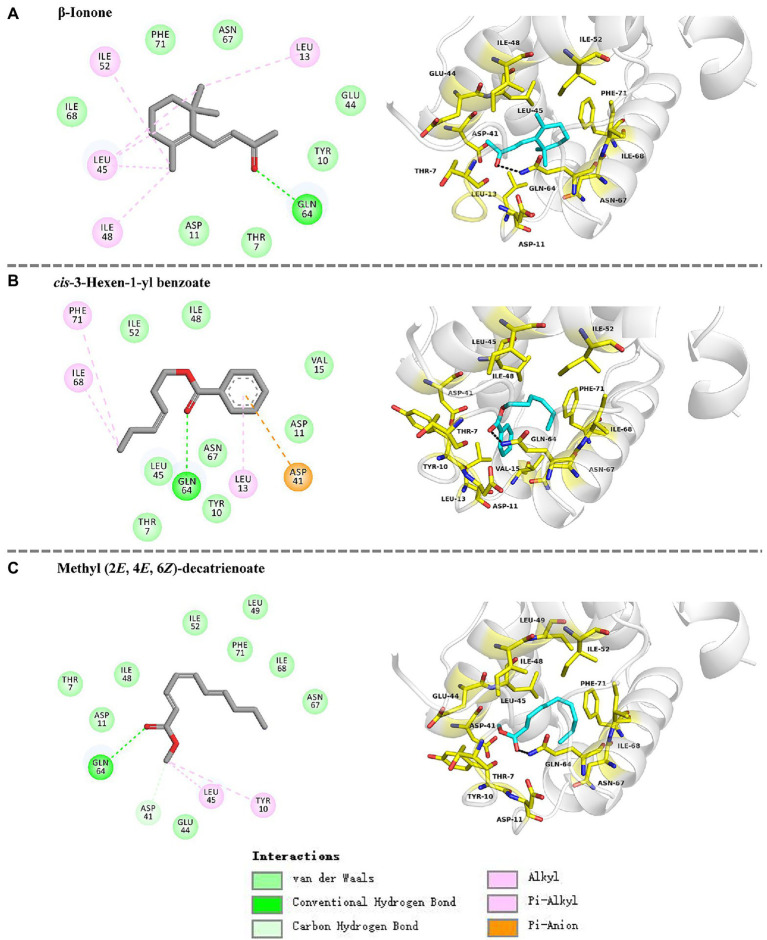
Molecular docking of HhalCSP15 with **(A)** β-ionone, **(B)** *cis*-3-hexen-1-yl benzoate, and **(C)** methyl (*2E,4E,6Z*)-decatrienoate.

The docking results showed that the ligands tightly bind to the HhalCSP15 pocket with negative energy values (Table 2). The 2D and 3D ligand interaction diagram is shown in Figure 5. The results indicated that different residues from the binding pocket participated in the recognition of distinct ligands. The HhalCSP15 amino acid residue Glu64 forms a hydrogen bond with all three compounds. Apart from hydrogen bond formation, the compounds exhibited van der Waals as well as hydrophobic interactions with HhalCSP15 (Figure 5; Table 2).

**Table 2:** Docking results for HhalCSP15 with different ligands.

Ligands	Binding energy (Kcal/mol)	Residues involved in hydrogen bond	van der Waals interactions	Hydrophobic interactions
β-Ionone	−7.2	Glu64	Thr7, Tyr10, Asp11, Glu44, Asn67, Ile68, and Phe71	Leu13, Leu45, Ile48, and Ile52
*cis*-3-Hexen-1-yl benzoate	−6.3	Glu64	Thr7, Tyr10, Asp11, Val15, Leu45, Ile48, Ile52, and Asn67	Leu13, Ile68, and Phe71
Methyl (2*E*,4*E*,6*Z*)-decatrienoate	−6.3	Glu64	Thr7, Asp11, Glu44, Ile48, Leu49, Ile52, Asn67, Ile68, and Phe71	Tyr10 and Leu45

### Electrophysiological Activities of Putative Ligands of HhalCSP15

**Figure 6:**
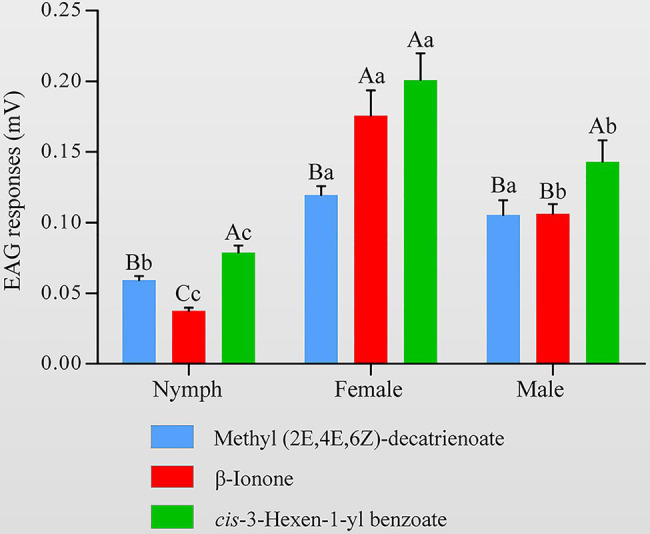
Electroantennography (EAG) activity of *Halyomorpha halys* antennae to different ligands of HhalCSP15. Different uppercase letters indicate significant differences among different chemicals, and different lowercase letters indicate significant differences among nymphs, females, and males (*p*<0.05).

To determine whether the HhalCSP15 ligands have biological activity, we measured the electrophysiological responses of nymph and adult *H. halys* to these three volatiles using EAG recordings. The results indicated that all three volatiles elicited electrophysiological responses in the antennae of both nymphs and adults (Figure 6). Adult *H. halys* showed significantly greater responses than nymphs. *Cis*-3-hexen-1-yl benzoate and β-ionone elicited significantly greater responses in females compared with males and nymphs. *EEZ*-MDT stimulated significantly greater EAG responses in adults compared with nymphs, but there was no significant difference between the sexes of adults. Of note, *cis*-3-hexen-1-yl benzoate elicited the highest EAG response among all ligands in both nymphs and adults (Figure 6).

## Discussion

Insect CSPs exhibit broad expression profiles both in chemosensory organs and non-chemosensory organs. In *H. halys* antennal transcriptome, 17 CSPs were identified and half of them had diverse expression patterns (Sun et al., 2020). In southern green stink bug *Nezara viridula*, 13 CSP genes were identified from antennae and mouthpart transcriptome, and only four genes were primarily expressed in antennae (Wu et al., 2019). The broad and diverse expression patterns of stink bug CSPs are consistent with their possible multiple roles in chemoreception, development, and other processes. HhalCSP15 is orthologous with NvirCSP4 (GenBank: QCZ25118.1, 81% identity) and both of them are specifically expressed in antennae, which suggests their conserved roles in olfactory perception. *NvirCSP4* was expressed roughly equally in both male and female antennae (Wu et al., 2019). Our data also reveal that no obvious difference was observed in expression levels of *HhalCSP15* between male and female antennae. In addition, *HhalCSP15* was also specifically expressed in nymph antennae. Thus, it is conceivable that HhalCSP15 is involved in the detection of odorants eliciting common stink bug behaviors, such as host location or intraspecific communication.

In fluorescent binding assays, 43 volatile compounds, including plant volatiles and *H. halys* volatiles, were selected as candidate ligands. Ligand-binding experiments demonstrated that HhalCSP15 has highly selective binding to volatile compounds. Some specific amino acids located in the hydrophobic cavities may be involved in the process of ligand binding in HhalCSP15 (Tomaselli et al., 2006). For example, in CSPsg4, I76 and W83 are involved in oleamide binding (Tomaselli et al., 2006); in CSP2, Y11 plays a key role in the binding of (*E*)-3,8-dimethyl-1,4,7-nonatriene (Li et al., 2021). Molecular docking analyses indicate favorable interactions between HhalCSP15 and its ligands. Glu64 forms a hydrogen bond with all ligands and could actively participate in forming the binding site of HhalCSP15. However, the specific binding sites that mediate the interactions between HhalCSP15 and ligands need to be investigated in future site-directed mutagenesis experiments.

β-Ionone and *cis*-3-hexen-1-yl benzoate are widely distributed in plants (Fraser et al., 2003; Simkin et al., 2004; Wei et al., 2011; Baldermann et al., 2012; Suckling et al., 2012). The binding experiments showed that the two compounds have strong binding abilities with HhalCSP15 and elicit an EAG response in both nymph and adult *H. halys* antennae. *EEZ*-MDT, which was identified as a binding ligand of HhalCSP15, is the aggregation pheromone of *Plautia stali*, which attracts stink bugs and is used as a lure in traps to monitor *H. halys* (Sugie et al., 1996; Aldrich et al., 2007; Morrison et al., 2017). *H. halys* does not emit *EEZ*-MDT though it may use *EEZ*-MDT as an indirect clue when searching for food plants (Funayama, 2008; Weber et al., 2017). These data further support a potential role of HhalCSP15 in *H. halys* host location. It was also found that HhalCSP15 could not bind with selected *H. halys*-derived volatiles, such as tridecane and *(E)*-2-decenal. *(E)*-2-decenal is an alarm pheromone in *H. halys* (Harris et al., 2015), and at least five *H. halys* OBPs showed high binding activities to it (Zhong et al., 2018). Thus, these findings also indicate that HhalCSP15 is not a pheromone binding protein of the stink bug and unlikely participates in the intraspecific communication for *H. halys*. However, gene editing and behavioral assays need to be further performed to verify the roles of this protein in the olfactory system of *H. halys*.

In conclusion, we report the antenna-specific expression as well as ligand binding capability of the CSP HhalCSP15 from *H. halys*, providing evidence for the possible olfactory roles of CSPs in the host-finding behavior of stink bugs. Although our results indicate that β-ionone and *cis*-3-hexen-1-yl benzoate are potential bioactive volatiles, further studies are necessary to confirm their behavioral activity as well as their possible applications for regulating the olfactory behavior of *H. halys*.

## Data Availability Statement

The original contributions presented in the study are included in the article/Supplementary Material, further inquiries can be directed to the corresponding author.

## Author Contributions

SW conceived and designed the research and wrote the manuscript. SW and ZW conducted all the experiments. ZW, AS, and FY analyzed the data. SS and YZ revised the manuscript. All authors contributed to the article and approved the submitted version.

## Conflict of Interest

The authors declare that the research was conducted in the absence of any commercial or financial relationships that could be construed as a potential conflict of interest.

## Publisher’s Note

All claims expressed in this article are solely those of the authors and do not necessarily represent those of their affiliated organizations, or those of the publisher, the editors and the reviewers. Any product that may be evaluated in this article, or claim that may be made by its manufacturer, is not guaranteed or endorsed by the publisher.

## Footnotes

^1^ http://primer3.ut.ee/

^2^ https://www.rcsb.org/

^3^ https://salilab.org/modeller/9.25/release.html

^4^ https://saves.mbi.ucla.edu/

^5^ http://vina.scripps.edu/

^6^ https://pymol.org/2/
