# Supplementary material for: Expression Profiles and Functional Characterization of Chemosensory Protein 15 (HhalCSP15) in the Brown Marmorated Stink Bug Halyomorpha halys
Source: Front Physiol. 2021 Sep 6;12:721247. doi: 10.3389/fphys.2021.721247 (PMC8450399; doi:10.3389/fphys.2021.721247)
Supplement: Supplementary file 1 [file Data_Sheet_1.PDF]

# Supplementary Material

CSP15

| | |
|---|---|
| 1 | ATGACAGACAAAATGATGCTGGTCCTTTCACTGTTGATGGTCTCTGTGGCCGCTGCTCTT |
| 1 | M T D K M M L V L S L L M V S V A A A L |
| 61 | CCTGCTGACACGTACACCACCAAGTACGACAACCTCGACGTCGGAGAGATTCTCAAGAAC |
| 21 | P A D T Y T T K Y D N L D V G E I L K N |
| 121 | GACAGGCTTTACCAGAAGTATAATGAGTGTCTGAGTAATACTGGAACATGTACGCCAGAT |
| 41 | D R L Y Q K Y N E C L S N T G T C T P D |
| 181 | GGCAAGGAGCTCAAAGATATATTGGGAGAAATAATAAAAACAGACTGCAAGAAATGCTCT |
| 61 | G K E L K D I L G E I I K T D C K K C S |
| 241 | GAAAAACAAAAGAAGAACATCGTTAAATTTCTGAAGCAAATTTTGGAGGAAAAACCAGAG |
| 81 | E K Q K K N I V K F L K Q I L E E K P E |
| 301 | GACTTCGTGAAGTTGGAGAAAATTTACGATCCAGACCAAGTGTTCAGGAAGAAATATGCT |
| 101 | D F V K L E K I Y D P D Q V F R K K Y A |
| 361 | TCTTAA |
| 121 | S * |

**Figure S1**. Sequence characteristics of *HhalCSP15*. The predicted signal peptide is highlighted in brown. Four conserved cysteine residues are shown in red.

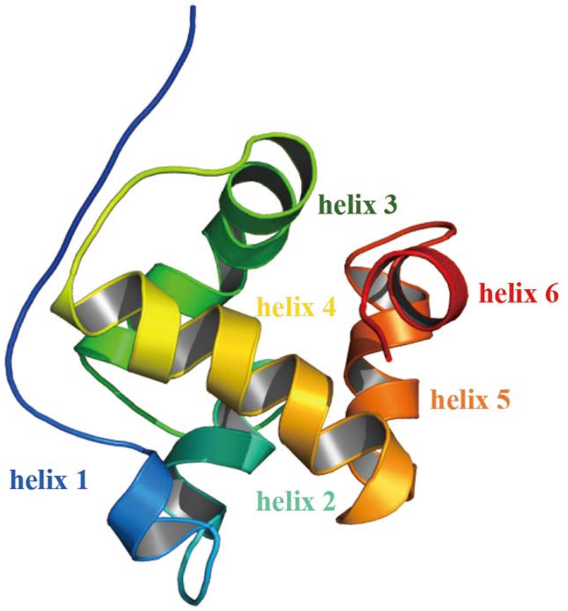


**Figure S2.** Three-dimensional (3D) structure of HhalCSP15. Helices α1–α6 are labeled.

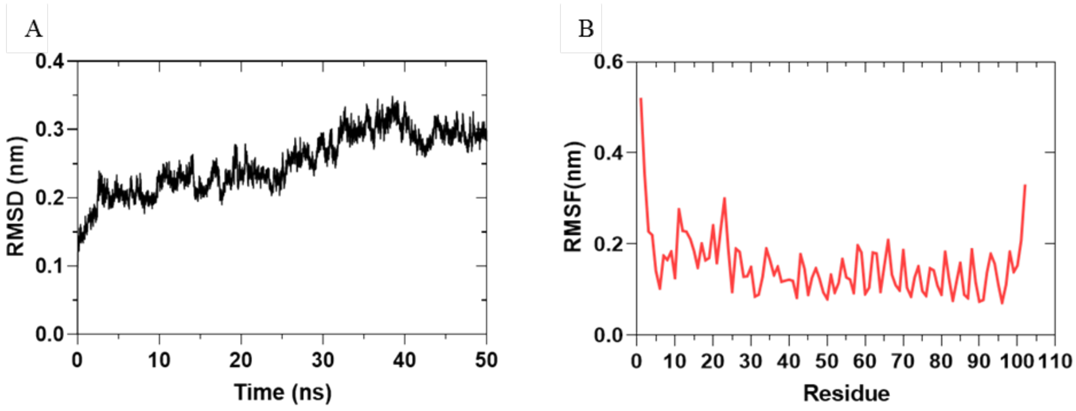


**Figure S3.** MD simulation results of HhalCSP15. (A) RMSD plot showing the conformational changes of the protein till 50 ns. (B) RMSF plot showing residue fluctuations for 50 ns.

PROCHECK

# Ramachandran Plot

## saves

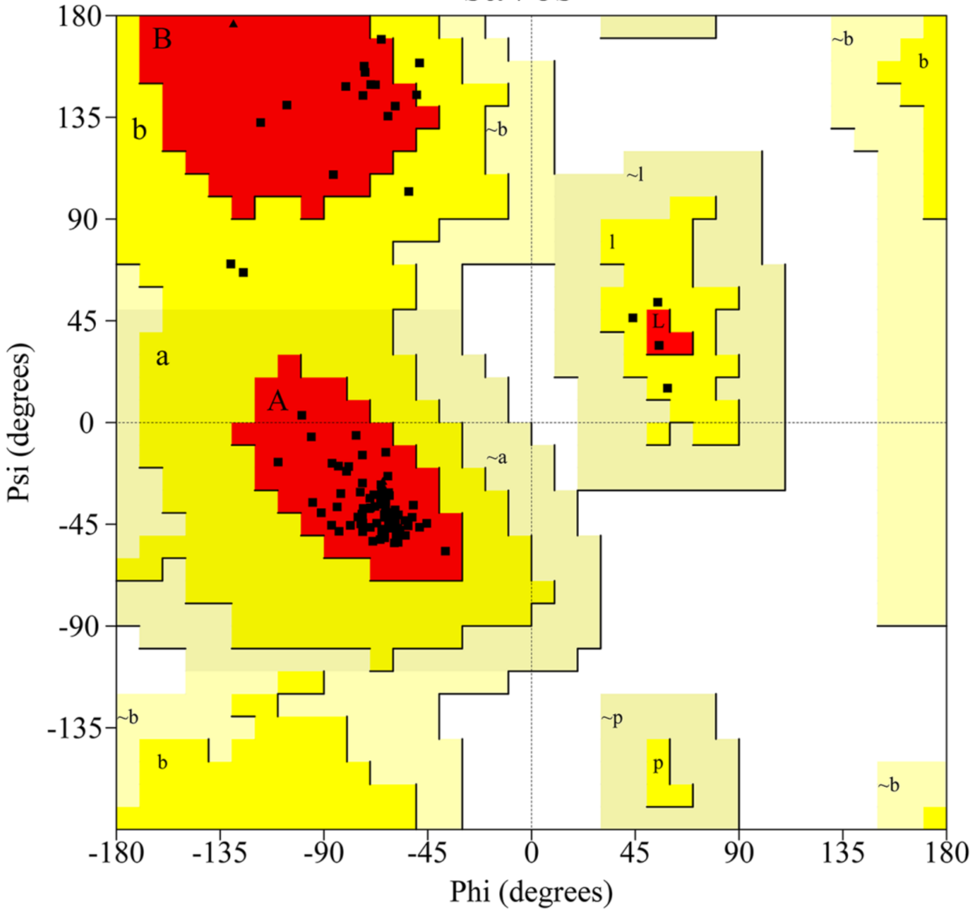


Plot statistics

| | | |
|---|---|---|
| Residues in most favoured regions [A,B,L] | 86 | 93.5% |
| Residues in additional allowed regions [a,b,l,p] | 6 | 6.5% |
| Residues in generously allowed regions [~a,~b,~l,~p] | 0 | 0.0% |
| Residues in disallowed regions | 0 | 0.0% |
| | ---- | ------ |
| Number of non-glycine and non-proline residues | 92 | 100.0% |
| Number of end-residues (excl. Gly and Pro) | 2 | |
| Number of glycine residues (shown as triangles) | 4 | |
| Number of proline residues | 4 | |
| | ---- | |
| Total number of residues | 102 | |

Based on an analysis of 118 structures of resolution of at least 2.0 Angstroms and R-factor no greater than 20%, a good quality model would be expected to have over 90% in the most favoured regions.

**Figure S4.** PROCHECK results from the predicted 3D model of HhalCSP15.

**Table S1** Primers used in this study

| Gene | Primer sequences | Primer use |
|---|---|---|
| *CSP15* | F: GAGAGATGACAGACAAAATGAT<br>R: TGATCACTTTGTTATAAAGC | Sequence verification |
| *CSP15* | F: ATGCTGGTCCTTTCACTGTTGAT<br>R: GAACACTTGGTCTGGATCGTAAA | RT-PCR |
| *EF 1-α* | F: GGCATTGGGAGATATCTTAACGG<br>R:TTGAAAGATCCTTTGCCCATCTC | |
| *CSP15* | F: ATGCTGGTCCTTTCACTGTTGAT<br>R: GGTAAAGCCTGTCGTTCTTGAGA | qRT-PCR |
| *Ubiquitin* | F: AGGCAAATAGGAATGACAGCGAG<br>R: GAGCATGGCAGTAATCCTTTCGA | |
| *EF 1-α* | F: CAATTATTGATGCCCCTGGTCAC<br>R: TACCGGCTTCAAATTCACCAGTA | |
| *CSP15* | F: CCATGGCTCTTCCTGCTGACACGTACAC<br>R: CTCGAGTTAAGAAGCATATTTCTTCCTGAAC | Heterologous expression |

The restriction enzyme sites are underlined.
